# Pathogenicity and virulence of monkeypox at the human-animal-ecology interface

**DOI:** 10.1080/21505594.2023.2186357

**Published:** 2023-03-10

**Authors:** Md. Mazharul Islam, Pronesh Dutta, Rijwana Rashid, Syed Shariq Jaffery, Ariful Islam, Elmoubashar Farag, Susu M Zughaier, Devendra Bansal, Mohammad Mahmudul Hassan

**Affiliations:** a Department of Animal Resources, Ministry of Municipality, Doha, Qatar; b Faculty of Veterinary Medicine, Chattogram Veterinary and Animal Sciences University, Chattogram, Bangladesh; c Department of Health Protection and Communicable Disease Control, Ministry of Public Health, Doha, Qatar; d Ecohealth Alliance, New York, NY, USA; e College of Medicine, QU Health, Qatar University, Doha, Qatar; f Queensland Alliance for One Health Sciences, School of Veterinary Science, The University of Queensland, Gatton, Australia

**Keywords:** Monkeypox, Mpox, clinical features, drivers, prevention and control, one health

## Abstract

Monkeypox (Mpox) was mostly limited to Central and Western Africa, but recently it has been reported globally. The current review presents an update on the virus, including ecology and evolution, possible drivers of transmission, clinical features and management, knowledge gaps, and research priorities to reduce the disease transmission. The origin, reservoir(s) and the sylvatic cycle of the virus in the natural ecosystem are yet to be confirmed. Humans acquire the infection through contact with infected animals, humans, and natural hosts. The major drivers of disease transmission include trapping, hunting, bushmeat consumption, animal trade, and travel to endemic countries. However, in the 2022 epidemic, the majority of the infected humans in non-endemic countries had a history of direct contact with clinical or asymptomatic persons through sexual activity. The prevention and control strategies should include deterring misinformation and stigma, promoting appropriate social and behavioural changes, including healthy life practices, instituting contact tracing and management, and using the smallpox vaccine for high-risk people. Additionally, longer-term preparedness should be emphasized using the One Health approach, such as systems strengthening, surveillance and detection of the virus across regions, early case detection, and integrating measures to mitigate the socio-economic effects of outbreaks.

## Introduction

Following the coronavirus disease-19 (COVID-19) pandemic, monkeypox (Mpox) is the latest in the line of zoonotic infection that is of global public health concern [1,2]. The World Health Organization (WHO) declared the disease a public health emergency of international concern on 23 July 2022 and proposed “mpox” as a synonym for the old term “monkeypox” on 28 November 2022 [3–5]. The infection is caused by the mpox virus, which has been identified among different species of monkeys and rodents, but the original reservoir host remains unknown [6]. After the first inception of mpox in humans in the Democratic Republic of the Congo (DRC) in 1970, it became endemic in Central and West Africa, mainly in the deeply forested areas [7]. Although, few human mpox cases were reported from outside Africa, who had travel and/or animal trade history with the endemic countries. Ever since 1970, the number of cases has been gradually increasing, with a noticeable spike observed after 2017 [8]. However, the virus emerged with a new dynamic in May 2022 and was mainly reported in non-endemic countries, more prominently in the American and European regions [9].

Since the 1970s, humans have acquired infection mainly in the rural and tropical rainforest areas of West and Central Africa through physical contact with an infected animal or host [10,11]. Human-to-human transmissions primarily occur in close contact in family and healthcare settings [12,13]. In addition, children and young individuals contract the infection when they come into close contact with infected wild animals during hunting and consumption of bush meat [11]. As such, an infected child is likely to pass and spread the infection to his/her siblings at a later point due to close contact in household settings [11]. It has been observed that the rate of infection is high among females than males, because the former tend to be primary caregivers at home and hospitals, thus they are at high risk for contracting the virus [14,15]. In the 2022 outbreak, the majority of the cases were reported among middle-aged (20–50 years) men who had sex with men (MSM) and/or contact with symptomatic or asymptomatic men [16–18]

Prevention of any zoonotic disease requires a dynamic approach to control the pathogen at the human-animal-ecosystem interface [19]. To this end, the One Health approach is a holistic method of managing and mitigating emerging infection-associated risks with a high chance of success [20,21]. “One Health is a collaborative, multisectoral, and transdisciplinary approach – working at the local, regional, national, and global levels – to achieve optimal health outcomes recognizing the interconnection between people, animals, plants, and their shared environment” [22]. In recent years, the One Health approach has been successfully practiced for the investigation and surveillance of emerging zoonotic diseases in several countries, including Qatar [23–25]. Such an approach is essential to understand the mpox virus and reducing its global health threat [26]. In this review, we explore the current knowledge of the virus, its distribution, possible drivers and dynamics of transmission, pathobiology and clinical features, and its ecological and epidemiological characteristics. Furthermore, we discuss the relevance of the One Health approach implementation to identify the possible challenges to reducing the global threat of mpox.

## The mpox virus, its ecology, and evolution

Poxviruses are double-stranded DNA viruses, enveloped with high molecular weight [27]. The mpox virus belongs to the *Orthopoxvirus* genera under the family *Poxviridae* and subfamily *Chordopoxvirinae*. There are around 18 species under the genera *Orthopoxvirus*, some of which are zoonotic. Mpox is a broad host range zoonotic virus that infects many species of mammals, including humans [28–30].

Although mpox endemic regions have been identified, however, the source of the virus remains unknown [31]. The understanding of the nature of the sylvatic cycle of the virus is also not yet established [31]. In addition, there is no obvious seasonal pattern for the disease, although the endemic regions are characterized by humid tropical evergreen rainforests. Moreover, environmental variables, such as annual precipitation, temperature, Net primary productivity of ecosystem, evapotranspiration, soil moisture, and pH are significant for the occurrence of mpox across these regions [32,33]. Also, high mean annual precipitation and low elevations were more significant with mpox occurrence [31].

The virus is genetically divided into two distinct clades, the Central African or Congo Basin clade and the West African clade [34–36]. Both are clinically and geographically different from each other. The West African clade originated in between the Niger and the Cross rivers, while the Congo Basin clade was detected in the south of the Sanaga river [37]. These rivers have been considered biogeographic barriers for natural host species of mpox transmission and resulted in genetic differentiation of the virus [37]. The West African clade is further divided into two subgroups: one isolated from Nigeria, and the other consists of isolates from Liberia, Ivory Coast, and the United States (US) [38]. The genome size of the West African clade (197,566–197,792 bp) is larger than the Congo Basin clade (196,850–196,959 bp) [39]. Furthermore, the reproduction number is estimated to be 0.6–1 [38,40], and the case fatality rate is reported to be higher in Congo Basin clade than in the West African clade [34,40]. Most of the human mpox cases reported in the Central African Republic (CAR) [41], DRC [42], and South Sudan [15] were Congo basin clade [36]. Besides this clade, the outbreak of 2003 in the US [43] and 2017 in Nigeria [44], Sierra Leone [45], Israel [46], and Singapore [47] were related to the West African clade. Initial analysis of the viral genome sequence of the first three identified positive cases in 2022 belong to the West African clade [48], which is closely similar to the previous imported case in the UK, Israel, and Singapore from Nigeria [44,49].

## Mpox in humans

Since the first reported case of human mpox on August 1970 in DRC [50], 37675 cases have been reported until May 2022 in several countries in Africa, America, Asia, and Europe [9,18]. Over 90% of these cases were reported within the Congo basin, with the highest number recorded in the DRC, followed by Ivory Coast, Congo, CAR, and Sierra Leone [51,52]. Ghana is the only endemic country where only animal cases were reported before 2022. However, several non-endemic countries such as Benin, South Sudan, Indonesia, Israel, Singapore, the United Kingdom (UK), and the US have reported cases before the 2022 outbreak (). Markedly, the US is the only non-endemic country that reported both human and animal cases several times. The first human mpox case outside the African region was reported in 2003 in the US [35,36], having a record of close contact with ill pet prairie dogs imported from Ghana [35]. In September and October 2018, three human cases were identified among international travellers from the UK and Israel who had a travel history to Nigeria [12]. Nigeria is one of the endemic countries for mpox in Africa that spread the infection to Israel, Singapore, the UK, and the US between 2019 and 2021 [53–55]. In South Sudan, the first human case was identified in 2005 and was not linked with any previous case or endemic region [15,56], suggested as an occasional or sporadic case that was introduced from local, putative animal reservoirs [56].

During the epidemic period from 2 May 2022 to 19 January 2023, 84716 laboratory confirmed cases of mpox were reported, along with 80 death in a total of 110 countries/territories worldwide, which are mostly non-endemic for mpox [9]. The American region showed the highest number of cases (68%), followed by Europe (30%), and the rest of the world (2%). It was observed that Nigeria was the epicentre of the mpox epidemic in 2022 as cases spread due to travel to this country [57]. Later, the cases spread among the family cluster, hospital outbreak, and persons who maintained physical/sexual relationships [58]. In addition, the endemic countries, such as Cameroon, CAR, DRC, Congo, and Nigeria showed several cases of mpox in 2022, with Nigeria having the highest number of cases [9]. The first human mpox case in Ghana, which was occurred in 2022 had a travel history to the US [59]. However, the South African case in June 2022 did not have any travel history and/or close contact with a positive case [60].

## Mpox in animals

**Figure 1.**
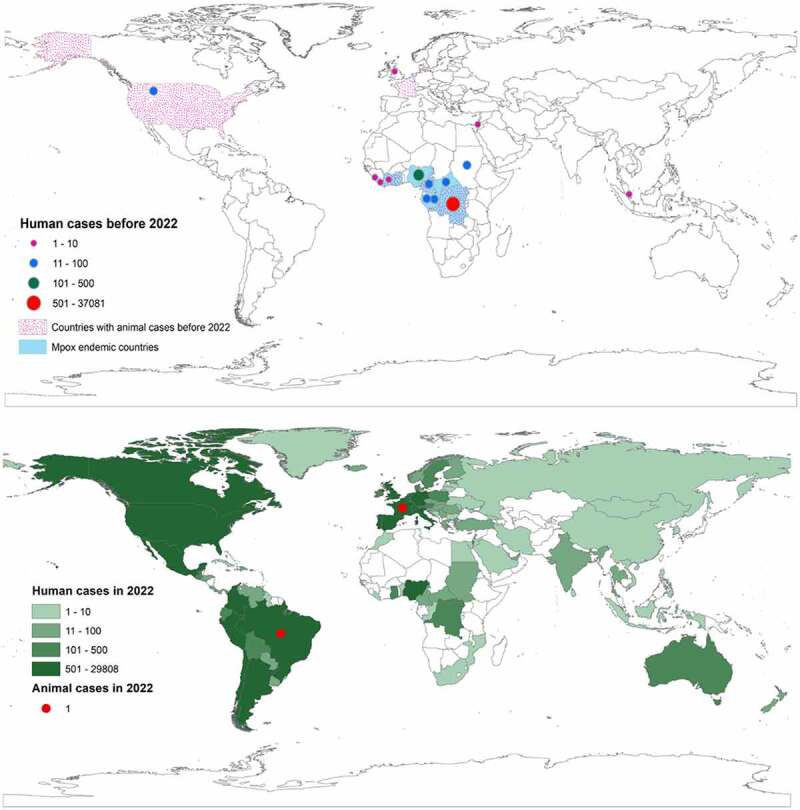
Global distribution of mpox at the human-animal interface. The upper global map shows the outbreak distribution countries before 2022 and the lower global map shows the outbreak distribution countries in 2022.

The first animal case of mpox was detected in a captive cynomolgus macaque (*Macaca fascicularis*) at a research institute in Copenhagen, Denmark, in December 1958, which was imported from Malaysia [61]. Subsequently, the virus was also detected among animals in natural and artificial habitats of different countries of the world (Table 1 and Figure 1). The first isolate of the mpox virus from a wild animal was detected in a rope squirrel (*Funisciurus anerythrus*) in DRC in November 1985 [74]. Another isolate from the wild animal was identified from a dead sooty mangabey (*Cercocebus atys*) in Taï National Park, Côte d’Ivoire, in March 2012 [66]. Rope squirrels in the natural settings of DRC were identified with 24.7% seropositivity [75]. Based on the field evidence, rodents including rope squirrels, sun squirrels, and non-human primates in DRC have been suspected as reservoirs of the virus [1]. The majority of the reported mpox in animals were from the US [62–65,71,72] and Central African countries, including the Ivory coast, DRC, and Cameroon [32,67,68,70,73]. Although imported animals from Malaysia, India, and Singapore were detected positive for mpox, the virus had not been detected among the local animals in these countries [65]. In August 2022, two cases of human-to-dog transmission were reported in France and Brazil [76,77].

**Table 1.** Reported non-human primate, rodent, and other species positive with mpox either by antibody to the virus, nucleotide detection, or virus isolation before 2022.

Family	Species	Reference
**Order: Primates**		
*Cercopithecidae*	*Cercocebus atys, Macaca fascicularis, Macaca mulatta*	[61–66]
*Cercopithecidae*	*Piliocolobus badius, Chlorocebus aethiops, Cercopithecus ascanius, Cercopithecus hamlyn, Cercopithecus petaurista*	[32,67–69]
*Callitrichidae*	*Callithrix jacchus* (*Hapale jacchus*)	[69]
*Cebidae*	*Saimiri* spp.	[69]
*Hominidae*	*Pan troglodytes, Gorilla gorilla, Pongo pygmaeus*	[65,69,70]
*Hylobatidae*	*Hylobates lar*	[69]
**Order: Rodentia**		
*Cricetidae*	*Allocricetulus* spp., *Cricetus* spp.	[71]
*Chinchillidae*	*Chinchill*a spp.	[71]
*Dipodidae*	*Jaculus* spp.	[71]
*Heterocephalidae*	*Heterocephalus* spp.	[71]
*Gliridae*	*Graphiurus* spp.	[72]
*Muridae*	*Rattus* spp., *Gerbillus* spp.	[71]
*Nesomyidae*	*Cricetomys* spp.	[72]
*Sciuridae*	*Funisciurus anerythrus, Funisciurus bayonii, Heliosciurus* spp., *Cynomys* spp.	[32,71–73]
**Order: Pilosa**		
*Myrmecophagidae*	*Myrmecophaga* spp.	[69]
**Order: Eulipotyphla**		
*Soricidae*	*Crocidura* spp.	[8]

## Mpox in environment

The mpox virus has not yet been detected in the natural environment. However, an experimental study showed the virus is transmissible through air, excretion of animals such as vomit, faeces, and skin scarification [11]. Moreover, the virus can survive in the dead organs of the rope squirrels for at least 7 hours at ambient temperature [78] and can spread through environment samples. Recently, Atkinson and colleagues (2022) have found the virus on environmental surfaces nearby, such as mattresses and sheets, towels, mobile phones, door handles, and sofas used by the patient [79].

## Possible drivers of transmission

**Figure 2.**
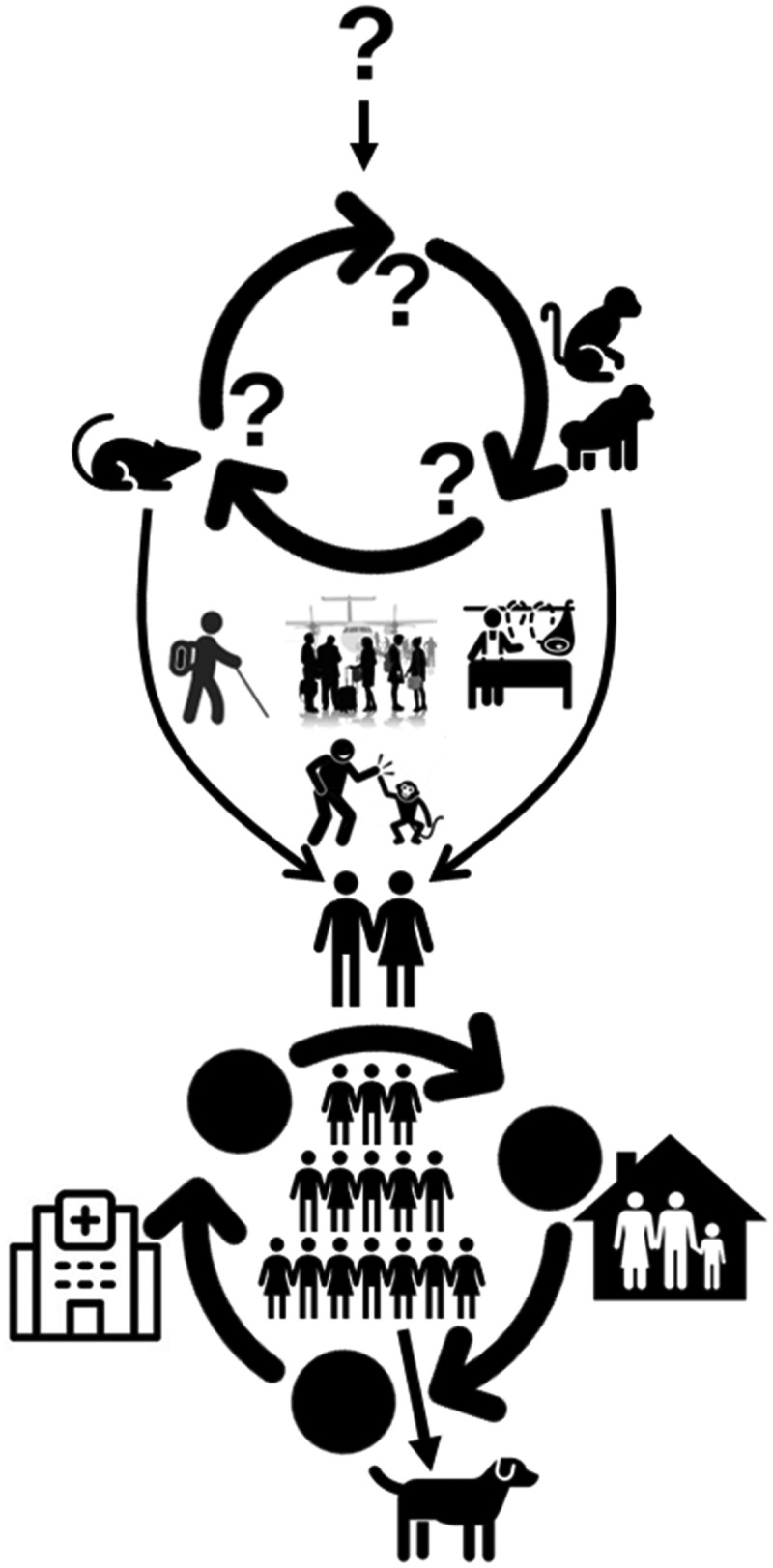
Origin and transmission dynamics of mpox. The origin of the virus or its natural reservoir is unknown. Rodents and non-human primates are susceptible to the virus, and humans can get the infection if get contact with infected humans, animals, or the environment when travelling to endemic countries, handling infected animals during bushmeat consumption or business, or for other purposes. Human-to-human transmission is possible in close contact, nosocomial, or family settings. There are recent reports of human-to-dog transmission.

The mpox virus naturally occurs in wild rodents and non-human primates of the forest area of West and Central Africa (Figure 2). Humans can get the infection through direct or indirect contact with infected humans, animals, or natural hosts. Based on the historical, cultural, political, economic, and state-citizen relations, and in conjunction with the experience from previous and current outbreaks and responses, several drivers may play an important role in mpox transmission.

### Trapping, hunting, and bushmeat consumption

The virus has been circulating in different animal species in central and western Africa [80]. Mpox infection in humans most commonly occurs in the endemic regions, particularly in small villages bordering the forests where they trap and hunt small wild animals like squirrels and take them back to their houses for consumption [11]. In an epidemiological investigation, 64.5% of patients had contact with monkeys at the presumed time of infection, and 11.8% had contact with both squirrels and antelopes [10].

### Travel and mass gathering

Mpox was detected in the UK, Israel, and Singapore in 2019 among travellers who had travel histories to Nigeria [12]. In 2022, the outbreak was also linked with a traveller who travelled from the UK to Nigeria [57]. During travel and mass gatherings, travellers may have contact with the mpox virus through a positive human or animal individual and acquired the infection [11]. The mpox epidemic in 2022 was primarily the result of travel and mass gathering in the Canary Islands, Spain for a festival [81].

### Animal trade

Animal trade by the importation of animals from the mpox endemic countries to the non-endemic countries is one of the drivers of mpox transmission. A report investigated that 71 people in the Midwestern US were infected in a multi-state outbreak caused by prairie dog distributors [35]. The distributors had a record of importing an infected rodent (Gambian giant rat) as a pet from Ghana in 2003, which subsequently infected humans in the US [35].

### Climate and ecological change

Humid and lowland evergreen tropical forest areas of Congo Basin, Central Africa, Guinea, Nigeria, Ivory coast, Sierra Leone, Ghana, and Tongo are considered potential distributional areas of mpox virus [31]. Mean annual precipitation is also a key factor in mpox transmission. These endemic areas have seen dramatic geographical and environmental changes over the last 30–40 years, mostly due to human population expansion, continuous deforestation, agricultural extension, urbanization, and climate changes [82]. Such changes alter the dynamics of human and animal interactions, where animals find themselves seeping into the human populations which in turn increases the risk of novel pathogens [8,83]. For example, the presence of rope squirrels in forest areas rich with the oil palm tree, the main food source for squirrels, is significantly important as a risk factor [84].

### Microbial evolution

The Congo basin clade is relatively less pathogenic compared to the West African clade, which spread to non-endemic countries. Though there are some ecological differences associated with human mpox between West Africa and Congo Basin but these dissimilarities could simply reflect the difference in habitat availability between the two regions [31]. Furthermore, the West African clade split into two branches, which may be behind the global transmission in recent years [85].

### Hospital settings

Several nosocomial outbreaks suggest that healthcare workers are a high-risk group for mpox. It may be due to incorrect or delayed confirmation of mpox cases, and lack or inappropriate use of personal protective equipment (PPE) [12]. In addition, the poor health infrastructure, lack of isolation facilities, and limited resources for diagnosis, and treatment of suspected cases with negative results may lead to transmission to a healthy person.

### Household transmission

Mpox has been documented with person-to-person transmission within the families or those who live in the same housing facilities [86]. When the inter-human transmission is 65.7 % with the human contact cases, 95.2% of them are intra-household [8]. Risk factors of acquiring mpox in a household included not practicing home isolation, sleeping in the same room or bed, and sharing home utensils among healthy and clinically sick individuals [86].

### Immunity

Smallpox vaccines prevent mpox with 85% efficacy and help to reduce disease severity [87–90]. Vaccination against smallpox has been stopped after declaring its eradication in 1972. Smallpox vaccine immunity, i.e. herd immunity effect gradually declined. In 2016, a survey found that only 10.1% of the total Nigerian population has antibodies against smallpox [91]. Additionally, before the 2017 outbreak, the estimated population immunity in Nigeria was only 2.6% in 2016 and the individual level immunity decline 1.29% per year. The decline in herd immunity for smallpox may be a contributor to the current mpox spread as cross-protective immunity is also reduced. As the number of non-vaccinated persons is increasing, combined with the gradual decline of immunity against smallpox, people are now more susceptible to mpox [91].

### Human behaviour

Mpox virus has been detected in semen [92] and can transmit through sexual contact with a person who has an active rash [8]. Most of the cases during the 2022 outbreak had a history of sexual contact, which is characterized as MSM [17,93].

## Pathobiology and clinical features in humans

### Pathobiology

**Figure 3.**
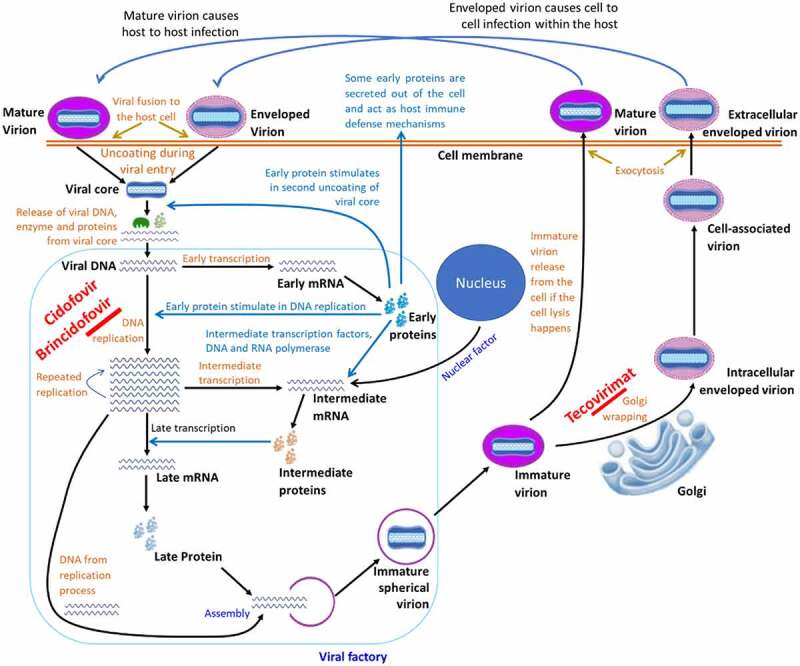
Cell-level replication cycle of mpox virus and mechanism of action of antivirals. The replication takes place in the cytoplasm of the host cell called the viral factory, following entry of a mature virion by micropinocytosis/fusion and enveloped virion by fusion method. After entry, the virion gets uncoated and the viral genome, protein, and enzyme are released to the host cytoplasm. The protein and enzyme initiate the replication process and prevent the cell defense to prevent replication. After a series of early, intermediate, and late phases of genomic replication, viral elements are assembled to form an immature virion, which later converts to a mature virion. The mature virion then gets a secondary membrane from the trans-Golgi network to form enveloped virion. The antiviral drugs “Cidofovir” and “Brincidofovir” prevents viral replication, whereas “Tecovirimat” prevents the envelope wrapping in Golgi.

**Figure 4.**
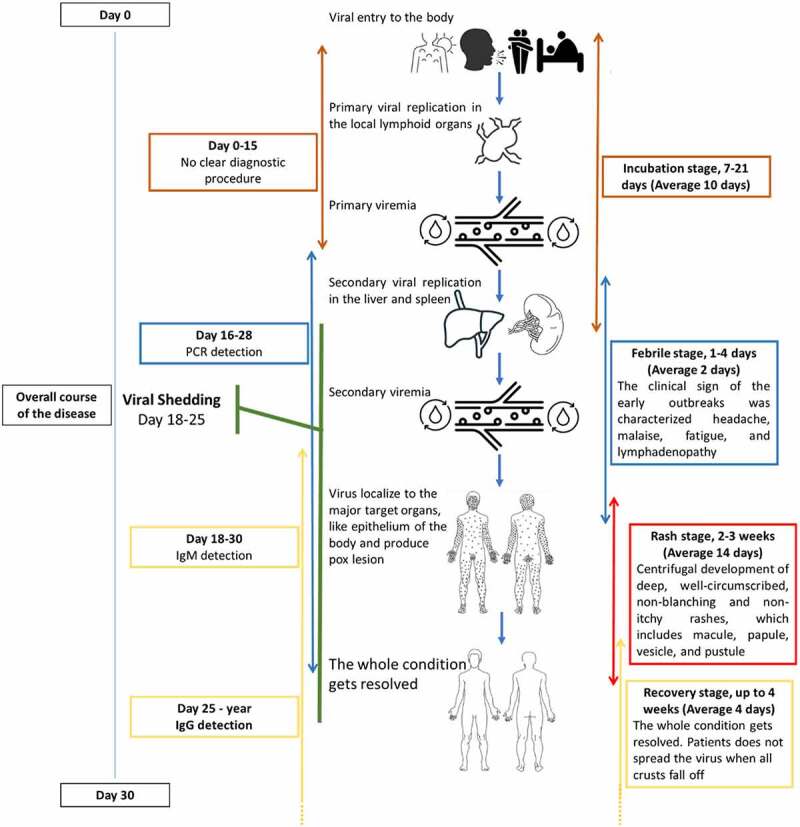
Pathogenesis of mpox virus in humans. After entry into the body, the virus replicates at the inoculation site and in the local lymph nodes. Primary viremia leads the viral spread towards other organs, like the liver and spleen, and replicates. Early clinical features start at this stage. When the secondary viremia takes place and the virus is transferred towards the cutaneous parts of the body, then mucosal and other lesions develop [98]. The molecular diagnosis is possible from the stage of primary viremia until the pustule formation. The antibody IgM can be detected when the secondary viremia starts till the lesions are resolved, whereas the IgG can be detected from the third week of infection till a year depending on the patient’s immune status. Viral shedding can happen with the beginning of clinical symptoms; however, the risk period of viral shedding is the vesicular and pustular stages.

The pathobiology of human mpox is nearly similar to that of smallpox, although the basic case reproduction number (R_0_) of smallpox is 6 [94], whereas it is 1.29 in the case of mpox [95]. As a zoonotic virus, primary transmission occurs through contact with infected animals. The viral inoculation to the body takes place through the respiratory tract (oropharynx or nasopharynx) and person-to-person transmission (intradermal). There is no known host cell receptor(s) and viral receptor-binding protein(s) for poxviruses. Many glycosaminoglycans, such as heparin sulphates, chondroitin, and laminin contribute to their attachment to cells [96]. A pox virion is minimally dependent on the host cell for its replication. After entry to the host cell, the virion undergoes a series of replication processes called first, intermediate, and late replication [96,97]. A detail of the mpox viral entry to the host cell, replication process, and exocytosis of the new virus is shown in Figure 3. The mechanism of mpox pathobiology, clinical courses, and viral shedding is presented in Figure 4.

### Clinical features

The clinical features include the cutaneous, gastrointestinal tract, and respiratory tract involvement along with other systemic illnesses [98–101]. The clinical sign of the early outbreaks was characterized by febrile condition with headache, malaise, fatigue, and lymphadenopathy, followed by centrifugal development of deep, well-circumscribed, non-blanching, and non-itchy rashes [102]. The rashes mostly develop on the face and extremities (including palms and soles), which may later spread towards the rest of the body including mucous membrane, buccal mucosa, conjunctivae, neck, trunk, palms, foot, soles, perineum, and penile shaft [99]. The number of rashes varies from a few to thousands and the size from 2 to 10 mm. The rash burden is classified into benign (5–25), moderate (26–100), grave (101–250), and plus grave (>250) types based on lesions [43]. After 2 to 4 weeks, the lesions evolve through macular, papular, vesicular, and pustular phases. Some rashes become crusting after black umbilication [52]. The consequences of infection range from mild to severe and fatal [102]. Lesions remain in the pustular phase for 5 to 7 days, then crusts form and desquamate by 7 to 14 days. The whole condition gets resolved within 4 weeks after the revealing of the symptoms. Patients do not spread the virus when all crusts fall off [98].

Secondary complications on the rashes can happen, such as bacterial superinfection, corneal infection, permanent scarring, bronchopneumonia, sepsis, septic shock, cellulitis, respiratory distress, encephalitis, dehydration, which may complicate the case, delay of recovery and sometimes lead to death [103]. Some adverse complications including vomiting and diarrhoea, corneal scarring, and encephalitis have been recorded in severe cases [43,100,104,105]. Abortion with cutaneous maculopapular lesions involving the skin of the head, trunk, and extremities, particularly the palms of hands and soles of feet of the foetus has been reported in pregnant infected women [88,106]. Clinical indications of the present 2022 epidemic emerge mostly in the genital area (perianal, scrotum, and lining of the penis) as whitish-coloured lesions that developed with the development of a central crust. In the clinical stage, papules with identical characteristics are also seen on the trunk and limbs [48]. The case fatality of human mpox disease in the previous outbreaks has been estimated at up to 11% [36,102], but in the present outbreak 3–6% were reported to die [16,107].

### Pathology

Gross pathology of the disease in humans includes encephalitis, peripheral and generalized lymphadenopathy, enanthema and exanthema (rashes inside and outside the body), haemorrhagic pustules, petechiae and purpura, development of pitting scars, hypopigmentation at scar, vesiculation, reticular degeneration, epidermal and lung tissue necrosis, focal and diffused oedema mainly at the facial and periorbital site, haemorrhagic and ulcerative oral mucosa, tonsillitis, laryngitis, pharyngitis, pleuritis, bronchopneumonia, diffuse pulmonary consolidation, dehydration, hypoproteinemia, hypoalbuminemia, low haematocrit level, colitis, and gastritis [43,52,54,99,104,108].

Microscopic findings for human mpox consist of the presence of poxviral antigen in the skin, oral mucosa, larynx, trachea, thymus, lymph node, spleen, lung, oesophagus, liver, stomach, intestine, and genitalia [43,100,104,108,109]. Later on, hypoxaemia, necrotizing lymphadenitis, tonsillitis, splenitis, oesophagitis, necro-ulcerative gastroenteritis, fibrinonecrotic bronchopneumonia, and necrotizing hepatitis was observed in very rare cases [43,87,90,100,104,108,110]. Additional findings are the absence of demyelination in acute encephalitis and development of cellulitis or sepsis [99,104]. Epidermal hyperplasia can develop along with diffuse, mixed, superficial, or dermal neutrophil exocytosis, leukocytosis, lymphocytosis, plasmacytosis, and mild to moderate thrombocytopenia or reduced platelet counts, infiltrate of dyskeratotic keratinocytes, episodic eosinophils and in the epidermis intracytoplasmic inclusion bodies consistent with Guarnieri bodies [46,54]. Other pathological features include elevated transaminase levels, elevated alkaline phosphatase levels, anaemia, hypoalbuminemia, and low blood urea nitrogen level [43,46,54].

## Diagnosis

### Case definition

The following case definitions are essential in understanding and diagnosing mpox defined by WHO, UK Gov, and CDC.

- **Infection period**: “The period beginning with the onset of the case’s first symptoms and ending when all scabs have fallen off” [111]
- **Contact: “**A person who has been exposed to an infected person during the infection period, or with a probable or confirmed case of mpox” [111]
- **Exposures**: “(1) Direct physical contact (such as touching, hugging, kissing, intimate or sexual contact), (2) contact with contaminated materials such as clothing or bedding, including material, (3) dislodged from bedding or surfaces during handling of laundry or cleaning of contaminated rooms, (4) prolonged face-to-face respiratory exposure in close proximity, (5) respiratory exposure (i.e. possible inhalation of) or eye mucosal exposure to lesion material (e.g. scabs/crusts) from an infected person (6) health workers potentially exposed in the absence of proper use of appropriate PPE” [111].
- **Suspected case**: “New characteristic rash OR Meets one of the epidemiologic criteria and has a high clinical suspicion for mpox” [112]
- **Confirmed case**: “Demonstration of the presence of mpox virus DNA by PCR or Next-Generation sequencing of a clinical specimen OR isolation of mpox virus in culture from a clinical specimen” [112]
- **Possible case**: “Suspected case who probably had exposure with a positive case” [113]
- **Probable case**: “No suspicion of other recent Orthopoxvirus exposure (e.g. Vaccinia virus in ACAM2000 vaccination) AND demonstration of the presence of Orthopoxvirus DNA by polymerase chain reaction of a clinical specimen OR Orthopoxvirus using immunohistochemical or electron microscopy testing methods OR Demonstration of detectable levels of anti-orthopoxvirus IgM antibody during the period of 4 to 56 days after rash onset” [112]
- **Epidemiological criteria**: “Within 21 days of illness onset: Reports having contact with a person or people with a similar appearing rash or who received a diagnosis of confirmed or probable mpox OR Had close or intimate in-person contact with individuals in a social network experiencing mpox activity, this includes MSM who meet partners through an online website, digital application (‘app’), or social event (e.g. a bar or party) OR Traveled to a country with confirmed cases of mpox or where mpox virus is endemic OR Had contact with a dead or live wild animal or exotic pet that is an African endemic species or used a product derived from such animals (e.g. game meat, creams, lotions, powders, etc.)” [112]
- **Exclusion Criteria**: “A case may be excluded as a suspect, probable, or confirmed case if: An alternative diagnosis can fully explain the illness OR An individual with symptoms consistent with mpox does not develop a rash within 5 days of illness onset OR A case where high-quality specimens do not demonstrate the presence of Orthopoxvirus or mpox virus or antibodies to orthopoxvirus” [112]

### Laboratory diagnosis

Diagnosis of mpox is based on epidemiological and clinical features as well as laboratory confirmation. Behavioural investigation as well as the study of recent travel history and contact with any suspected and/or confirmed case are important for ascertaining the initial investigation of mpox. Laboratory tests such as reverse transcriptase polymerase chain reaction (RT-PCR) (conventional or real-time method), recombinase polymerase amplification (RPA), loop-mediated isothermal amplification (LAMP), and restriction-fragment-length polymorphism (RFLP) [101], viral culture, enzyme-linked immunosorbent assay (ELISA), western blot, immunohistochemistry, and electron microscopy are performed to diagnose any suspected cases. PCR is considered a gold standard in the diagnosis of the mpox, however, if the test is negative and the case is still suspected, then other test methods can be applied for diagnostic confirmation. Electron microscopy is generally used for the morphological identification of the virus in the lesion. The presence of mpox virus-specific antigen in the lesion biopsy was detected by an Immunohistochemistry test [114]. Though smallpox-vaccinated persons can give false mpox positive results by the immunological test hence, serology is more applicable in epidemiologic, retrospective, and post-infection surveillance [18].

The virus has been detected in different samples such as saliva, rectal swab, nasopharyngeal swab, semen, urine, and faeces of the infected person [92,115], therefore sampling should be performed according to the diagnostic test (Figure 4). The pustule and blister substances are commonly used samples for molecular detection, although viral nucleic acid can be detected in oral/throat swabs, blood, faeces, and urine with good sensitivity and specificity. The same sample can be used for viral protein detection using serological methods like western blot and enzyme-linked immunosorbent assay (ELISA) [116]. The viral RNA is detectable from the febrile to the rash stage. However, IgM was detected throughout the rash stage and IgG was distinguished after one week of onset of the rash stage till one year [101,117].

### Differential diagnosis

Mpox must be differentiated from bacterial infection, chickenpox, measles, medication-associated allergies, scabies, and syphilis. Mpox is characterized by lymphadenopathy, a relatively long prodromal period, centrifugal distribution of the rash, and slow progression of lesions. However, there is no lymphadenopathy, short prodromal period (1–2 days), the rash is centripetal in distribution, and the spread of the rash is faster (incubation period: 10–21 days and the symptoms typically last for 4–7 days) in the case of chickenpox. The prodromal phase of measles is considered relatively longer than mpox (3–5 days). The lesions of mpox are usually seen on the face, palm, and sole, whereas chickenpox lesions are mostly seen on the trunk and measles lesions start on the face and later can transfer to the hand and feet. Lymphadenopathy is always seen in mpox but does not notice in chickenpox and is rarely seen in measles [101,118].

## Clinical management

Currently, there are no specific treatments for mpox, however, it is usually mild and self-limiting. The clinical management of mpox is mostly supportive care with symptomatic treatment, which includes antipyretics, analgesics, and antibiotics [114]. Certain patients may require specific treatment, especially in case of severe conditions, immunocompromised, paediatric patients, and pregnant women. Some patients require maintenance of adequate fluid balance, haemodynamic support, and supplemental oxygen or other respiratory support. Another aspect of supportive care is the management of ocular infection/complications, which can lead to corneal scarring and/or loss of vision [114].

Some antiviral agents have shown some degree of efficacy against the mpox virus, including cidofovir, brincidofovir, and tecovirimat [18,114]. Detailed modes of action of these drugs for the treatment of mpox have not yet been studied, however, some basic functions of these drugs based on animal experiments and treatment of other human viral diseases have been explored (Figure 3) [119]. Cidofovir is an intravenous injection that blocks viral DNA synthesis by inhibiting DNA polymerase. Brincidofovir is applied orally, which conjugates with cidofovir. Tecovirimat is available in both oral and intravenous injection formats, which inhibits the activity of protein VP37, thus abnormal virion formation and cannot release from the host cell. Vaccinia immunoglobulin (Ig) provides passive immunity to the patient [18,119], that is why, in addition to antiviral treatment, plasma therapy (from healthy donors who received a vaccinia vaccine previously and developed a high level of anti-vaccinia antibodies) is also recommended. These antibodies can bind to the poxvirus and prevent it from infecting new cells. This type of treatment is commonly used to treat virological infections, such as COVID-19 [18,114,120].

## Prevention and control strategies

The main objective in the prevention and control of mpox should be to interrupt the multi-country outbreak and prevent virus transmission at the human-animal interface. It should go through infection prevention and control (IP&C), where vaccination should be considered as an additional measure [111,121].

### Infection prevention and control

Despite continuous efforts towards the development of effective therapy, other public health control, and preventive measures, such as active surveillance, early case detection, diagnosis, and care, should be used and prioritized. In addition, other interventions like contact tracing, self-monitoring by contacts, good hygiene practices, avoiding contact with animals or other suspected materials, and use of PPE should be used to reduce spread of the disease [122].

A national-level IP&C strategy and proper mpox patient handling guideline can prevent the nosocomial and family cluster transmission of the virus [2,89]. The healthcare workers, who work with suspected or confirmed mpox cases are suggested to use PPE [47,89] and should follow the routine monitoring and screening to prevent nosocomial infection of mpox. Female care providers at home and hospital settings should also wear PPE and take special precautions, as morbidity rates are higher amongst females [89,90]. Isolation of the patients and suspected case helps to reduce viral transmission. They must stay at home or in a government-provided quarantine facility for the incubation period (usually 21 days). Once mpox infected person recovered, it is recommended that the home should be sanitized before other family members and pets return to building [123].

Animals used as pets are at high risk of contracting the infection from their infected owners and their contacts. There are two recent instances of mpox virus transmission where pet dogs caught the infection from their owners in France and Brazil, as previously mentioned [76,77]. Therefore, it is suggested that infected persons avoid contact with domestic animals, pets, and wildlife to prevent spreading the virus at least 21 days after first contact with any positive cases or after PCR positive report [123]. The Centers for Disease Control and Prevention of the United States (CDC) recommends guidelines for the public to avoid contact with any Gambian giant rats or prairie dogs that seem to be sick [105]. Positive animals or the animals that had direct contact with a positive case may be necessary to isolate and care in a separate place, where a healthy and low-risk person will take care of them. However, currently vaccination is not recommended for animals [123]. Veterinarians must wear PPE during the treatment and wash their hands after contact with and treatment of animals. Frequent hands and face washing can reduce the chances of acquiring any viral infection, including mpox [124]. However, rules for limiting international travelling should be encountered if an outbreak occurs. Strategies should be developed for proper investigation, surveillance, prophylaxis, treatment, and routine vaccination in endemic areas. Bushmeat consumption, hunting, and trade should be tackled through mass awareness, media campaigns, legislation, and law enforcement [2]. Moreover, strict laws and strategies should be developed to prevent deforestation and ecosystem changes to prevent local extinction and invasive of foreign species to protect the natural ecosystem for prevention and control of mpox viral outbreak through wild species.

### Vaccination

A specific vaccine against mpox is yet to be established [125]. The smallpox vaccine had 85% efficacy against other zoonotic orthopoxvirus infections, including the mpox virus, and reduced the case severity and fatality [125–127]. Smallpox-vaccinated individuals have a 5.2-fold lower risk of getting mpox infection than unvaccinated individuals [89]. Therefore, smallpox vaccination for high-risk people is the first and foremost preventive strategy against mpox viral infection in humans, although mass vaccination against mpox is not recommended in the current multi-country outbreak [111].

There are several smallpox vaccines available [8,128] however, WHO recommended three specific smallpox vaccines: ACAM2000 (replicating vaccinia-based vaccine), MVA-BN (non-replicating vaccine), and LC16 (minimally replicating vaccine) can be used in response to the current mpox outbreak. MVA-BN is a two-dose vaccine recommended for pregnant women, breastfeeding mothers, children, and persons with immunosuppression therapies or atopic dermatitis. ACAM2000 is a single-dose vaccine, contraindicated for immunodeficiency individuals, pregnant, and infants as mild to moderate local and systematic adverse effect has been detected, such as myopericarditis and vaccinia. Similar to ACAM2000, LC16 is a single-dose vaccine unsuitable for severe immunodeficiency individuals, as it causes frequent mild to moderate adverse effects. However, vaccine-related severe adverse effects by LC16 are rare or not present, and no information on pregnancy, breastfeeding, and children [111].

Although evidence of mpox prevention is limited, and vaccine safety profiles vary by product, so it is essential to consider when deciding to apply a vaccine. Pre-exposure vaccine (PPV) is applicable only for high-risk persons, whereas post-exposure vaccine (PEPV) is indicative for close contact with infected persons. High-risk individuals are those who are MSM, and those who have multiple sexual partners. Other high-risk individuals are sex workers and health workers (involved in treatment, care, testing, and outbreak response of mpox). PEPV is recommended within 4–14 days of first exposure with a positive case in the absence of symptoms in the contact person. Vaccination may be less effective after 14 days post-exposure [111,129]. Children, pregnant women, and immunocompromised persons who have a chance of developing the more severe disease when infected with mpox are to be vaccinated as a priority [111].

Vaccination should follow a careful evaluation of risks and benefits, with informed decision-making between individuals and healthcare providers. People born before 1980 were usually vaccinated against smallpox. However, their immunity has gradually waned over time. Therefore, it is highly recommended that this group receive mpox vaccines at pre- or post-exposure levels [111]. Vaccinated persons should continue to follow the safety measures to protect themselves and others from infection [129]. People who had contact with a mpox case after the first dose, before the second dose, should receive their second dose as scheduled. In case of a limited vaccine supply, a national vaccination action plan must be prepared [111]. People who received the first dose of the vaccine should follow safety measures to reduce their exposure to mpox until 4 days after the second dose [129].

## Knowledge gaps

The transmission and dynamics of the virus remain unclear. Although African rodents and non-human primates are considered possible reservoirs of the virus, however, that is yet to be confirmed. The cases reported in South Sudan and South Africa did not have any direct contact with the positive case or international travel history. Therefore, it is yet to be established if the virus is presented among the wildlife reservoirs outside the endemic countries.

Mpox cases were sporadic in the endemic countries prior to 2017, but they increased in numbers shortly after that. This increase in numbers may be attributed to improved detection and response to disease, environmental and ecological distribution of the virus, an increase of dynamics among the reservoir hosts, or host immunologic issues, which is still not clear [8]. The molecular epidemiology of the virus is unknown, which is important to understand the virus and its virulence. There are multi-scalar drivers of shifting the disease across countries, how many risks are existing for further global spread? A specific vaccine against mpox is yet to be discovered [130]. The rate of asymptomatic infection is unknown, although they may be in a key role in transmitting the disease across countries [8]. There is a history of reinfection after 10 months of prior infection, thus, clarifying the immunity from natural infection is essential [8].

## Key priorities and research questions

The overall goal is to control the multi-country outbreak of the disease in the endemic and non-endemic countries. Mpox research is required on three specific approaches: (i) citizen science and social science consideration, (ii) implementation research, and (iii) One Health initiatives.

### Citizen and social science

Citizen and social science are instrumental in gaining an understanding of social dimensions of transmission, response to the current situation, and future prediction of any disease. Community engagement and awareness are essential to alleviate stress and stigma related to mpox and to identify the junction between real information and misinformation across social networks during an epidemic. There is a need for sustained mpox preparedness and response efforts with the support of related stakeholders and partners. Citizen science and social science considerations can play a critical role in awareness development and balancing the tension between real information and stigmatization. Social and behavioural change practices can be helpful to improve hygienic practices and implement realistic and feasible strategies to reduce mpox cases in endemic and non-endemic communities [8,131–133].

### Implementation research

It is essential to employ full range research on diagnostics, sequencing, therapeutics, and vaccine development. There is a need to improve patient care pathways, and identify and protect vulnerable healthcare staffs. In the case of therapeutics consideration, standardized data and clinical trial protocols are effective. The use of a case reporting form can help better in understanding the clinical picture of mpox in a locality. There is a diagnostic challenge, especially for the asymptomatic cases. The endemic countries need to adapt and strengthen laboratory testing capacities at a national and sub-national level. In addition, non-endemic countries need preparedness for the emergence of the human mpox epidemic. Many assays are available but there is a need of validating new assays. In addition, the sample of choice for confirming the diagnosis, the development of a biobank for biological samples with positive controls, and the establishment of mpox viral genome sequencing locally and globally to monitor the emergence of infecting clades. Adaptation and application of the IP&C program will protect health workers and nosocomial transmission. The current knowledge gap on mpox vaccination should be addressed [111], which includes research on vaccine safety, vaccine immunogenicity and effectiveness, vaccination options, citizen science on vaccination, and vaccination outcome [111]. In addition, research on specific therapy and current therapeutic effectiveness, including post-exposure prophylaxis and treatment should also be considered [134].

### One Health initiatives

**Figure 5.**
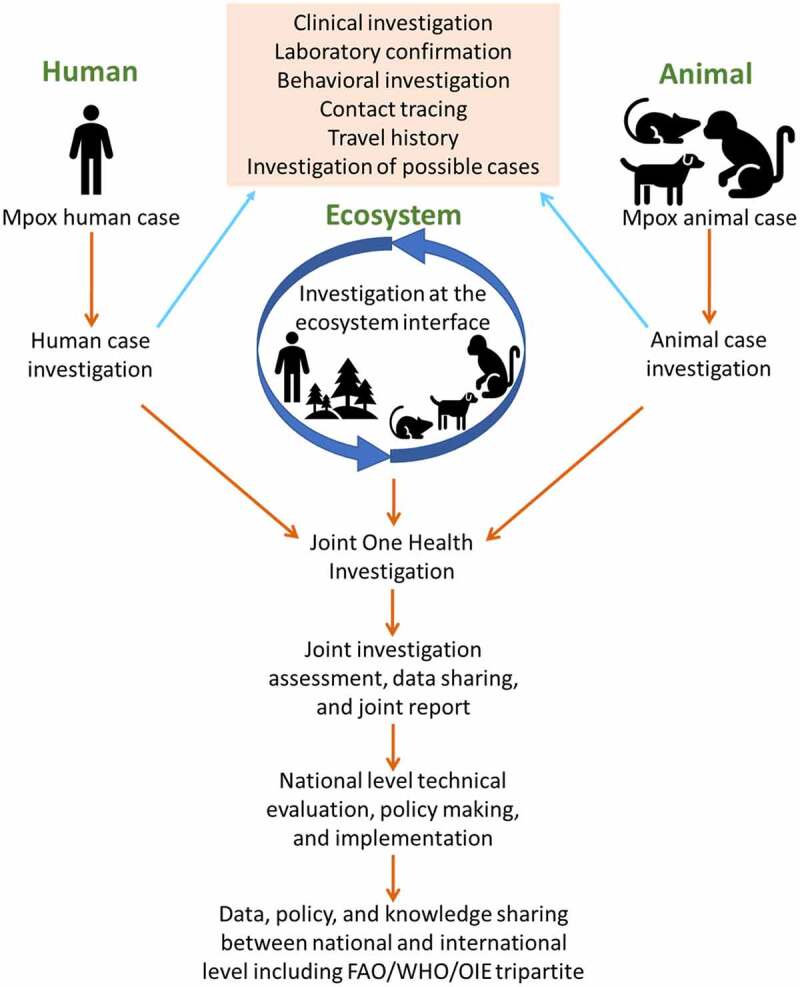
One health investigation of mpox at the human-animal-ecosystem interface.

A multidisciplinary One Health team needs to identify the animal source(s) and reservoir(s), future risks (behavioural, ecological, and socio-economic) of spillover as well as the possible modes of transmission at the human-animal-ecosystem interface (Figure 5) [135]. It is essential to test the seroprevalence of antibodies and antigens among high-risk humans and animals (wildlife, livestock, and pets), and identify the susceptible animals. A better understanding of mpox dynamics in bushmeat consumption, wildlife capture, transport, and trading will support the development of appropriate guidelines to prevent contracting the virus within said variables. Additionally, intensifying and strengthening current prevention strategies, such as surveillance, index case study, cluster investigation, contact tracing, isolation of patients, and examination of possible animals and environment will help in early case detection of cases. International organizations should allocate funding for research activities and publish new findings to promote advanced knowledge of mpox. A model of the disease can be developed following a global collaboration mechanism that will be effective for data and knowledge sharing and unified decision-making [8].

## Conclusion

Mpox remains a global health risk. The current review endeavoured to understand the mpox virus, its dynamics, and its pathological features in humans to propose a possible intervention strategy for the infection. The virus infects humans from unknown reservoir hosts and continues to spread mostly through human-to-human transmission. Human behaviour was observed to be a major factor in the 2022 global outbreak. Improvement of wildlife health surveillance, strengthening local health system capacities, and provision of training to the relevant personnel are essential components of being well-prepared. Increasing public awareness, as well as those of policymakers are crucial in developing proper mitigation programs. Longer-term preparedness should utilize One Health as a holistic system to detect, manage, and respond to this re-emerging health threat.
